# A comparison of three-dimensional stress distribution and displacement of naso-maxillary complex on application of forces using quad-helix and nickel titanium palatal expander 2 (NPE2): a FEM study

**DOI:** 10.1186/s40510-016-0131-3

**Published:** 2016-06-08

**Authors:** Avinash Kumar, Hajra Ghafoor, Arifa Khanam

**Affiliations:** Department of Orthodontics and Dentofacial Orthopedics, Al-Badar Dental College and Hospital, Gulbarga, Karnataka India

## Abstract

**Background:**

Our objectives are to analyse and to compare the stress distribution and displacement of the craniofacial structures, following the application of forces from quad-helix and Nickel Titanium Palatal Expander-2 (NPE2) using finite element analysis.

**Methods:**

Three-dimensional finite element models of young dried human skull, quad-helix appliance and NPE2 were constructed, and the initial activation of the expanders was stimulated to carry out the analysis and to evaluate the Von Misses stresses and displacement.

**Results:**

Both the models demonstrated the highest stresses at the mid-palatal suture, with maximum posterior dislocation. The second highest stress was recorded at the fronto-zygomatic suture. The pattern of stress distribution was almost similar in both the groups, but NPE2 revealed lower magnitude stresses than quad-helix. The only exception being quad-helix model showed high stress levels around pterygo-maxillary suture whereas minimal stress around pterygo-maxillary suture was noticed after NPE2 activation. The cusp of the erupting canine and the erupting mesiobuccal cusp of the second molar showed outward, backward and downward displacement signifying increase in their eruption pattern following maxillary expansion.

**Conclusions:**

Maxillary expansion using quad-helix and NPE2 can be used in posterior crossbite correction in cases where maximum skeletal changes are desirable at a younger age; it is furthermore effective in treating young patients with impacted or displaced teeth. Quad-helix and NPE2 produced acceptable forces for orthopaedic treatment even after being orthodontic appliances; their clinical application should be correctly planned as the effects of these appliances are largely age dependent.

## Background

Maxillary expansion treatments have been used for more than a century to correct maxillary transverse deficiency. The earliest common cited report was that of E.C. Angell published in Dental cosmos in 1860; however, the work was discredited at that time but the technique was generally accepted [1]. Slow maxillary expansion produces more physiologic response at the mid-palatal suture area. It produces less tissue resistance, since it delivers constant physiologic force in the suture and allows better bone formation, both these factors help to minimize the post expansion relapse [2].

Quad-helix has been evolved from the W arch of coffin by the incorporation of helical loops, two anteriorly and two posteriorly, which increased the flexibility and range of action of the appliance [3]. Arndt [4] introduced nickel titanium palatal expanders in the year 1993. Corbett [5] introduced Nickel Titanium Palatal Expander-2 (NPE2) in 1997. It generates optimal and constant pressure which is a consequence of nickel titanium’s shape memory and effects of transition temperature. It delivers a uniform, slow, continuous force for maxillary expansion, molar rotation, distalization and arch development.

Many research studies had been done in comparison between slow maxillary expansion and rapid maxillary expansion techniques, but very little had been done to compare differences between various techniques of slow maxillary expansion. Finite element analysis is an engineering method of calculating stresses and strains in materials, including living tissues [6]. Finite element method is closer to the clinical situation, and we accept that the qualitative behaviour of a dry skull does not simulate with a high degree of accuracy in the clinical situation, it is possible to indicate the way that the two maxillary halves separate during expansion and how the effect of the force influences the other craniofacial structures.

The main aim of this study was to analyse stress distribution and displacement of the craniofacial structures on the application of forces induced by quad-helix and NPE2 using finite element analysis.

## Methods

For creating a finite element model, a computer-aided design model was constructed from a dry young human skull of an approximate age of 12 years. Age estimation was carried out by dental eruption pattern. Human maxillary skull bone without mandible was checked for defects or discontinuity in the craniofacial anatomy. CT scan images of the maxillary bone were taken by SIEMENS SOMATOM Definition 64 (kVp120; mAs 290) in axial direction. Sequential CT images were taken at 0.5-mm intervals to reproduce finer and detailed aspects of the geometry. This scheme of model creation was intended to improve the anatomical accuracy over the previous methodologies where CT sections were taken at 1, 5, and 10 mm, respectively.

**Fig. 1:**
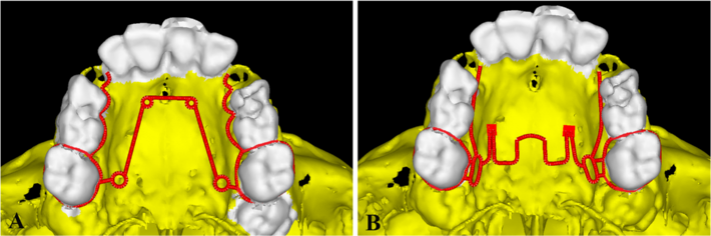
Schematic representation of the **a** quad-helix appliance model and **b** NPE2 appliance model

A total of 345 CT images in DICOM format were stacked over one another and converted to a finite element meshed model by the software Materialise’s Interactive Medical Image Control System (MIMMIC Version 18.0). Tetrahedron elements were used to mesh the skull. Quad-helix and NPE2 appliances were modelled by software ANSYS Design Modeller (Version 16; ANSYS Inc. Integrated Design Analysis Consultants) with beam elements (Fig. 1).

ANSYS Professional NLS (Version 16; ANSYS Inc.) was used to carry out the analysis. The total numbers of elements in the geometry were 864,650, and total numbers of nodes created were 247,119.

Thickness of the cortical bone was determined according to the study by Farnsworth et al. [7], thickness of periodontal ligament was 0.2 mm [8], and the thickness of the maxillofacial and mid-palatal sutures were 0.5 mm [9].

### Defining mechanical properties to the model [10–12]

The mechanical properties of the tooth, cortical bone, cancellous bone, suture, periodontal ligament, stainless steel and nickel titanium in the model were defined according to the experimental data in previous studies as shown in Table 1.

**Table 1:** Material property data representation

	Young’s modulus (Newton/mm^2^)	Poisson’s ratio
Tooth [10]	2.07 × 10^4^	0.30
Cortical bone [10]	1.37 × 10^4^	0.30
Cancellous bone [10]	7.9 × 10^3^	0.30
Suture [10]	7	0.40
Periodontal ligament [10]	50	0.49
Stainless steel [11]	2.1 × 10^5^	0.3
Nickel titanium [12]	44 × 10^3^	0.33

### Laying boundary conditions

The nodes of the mid-palatal suture element that were created in this study were placed on the symmetrical plane and were left unconstrained. Nodes along the foramen magnum and on the centre of the forehead were constrained in all degrees of freedom, with zero displacement and zero rotation [13]. The 3D coordinates were X, transverse plane; Y, sagittal plane; and Z, vertical plane. The midpoint of the lingual alveolar ridge of each tooth was used as a reference point to evaluate alveolar bone displacement. Positive values indicate outward, backward, and downward displacement on the X, Y, and Z planes, respectively.

In this study, a force of 350 g is used in case of NPE2 model as a 3-mm increment of expansion with NiTi palatal expander produces 350 g of force [5]. Quad-helix expansion appliance was activated to one molar width, i.e., around 8 mm. Chaconas [3] reported that an initial 8 mm expansion of quad-helix prior to cementation created approximately 14 oz (396.89 g) of force which is equivalent to 3.89 N, and therefore, 2 N of force was applied on each side. The displacements of maxillofacial complex and Von Misses stresses in different parts were studied. Von Misses is a criteria used in predicting the onset of yield in ductile materials.

## Results

The changes seen in the results were divided under two sections

- Displacements of various structures in all the three planes produced after the activation of both the appliances
- Von Misses stress distribution over the Naso-maxillary complex after the activation of both the appliances

All the maxillofacial structures in the transverse plane in both the models showed outward movement except for the lateral nasal wall and the inferior orbital rim. The lateral nasal wall and inferior orbital rim in both the groups showed inward, forward and downward movement. Sagittally, all the structure showed forward movement, except point A, ANS, PNS, maxillary tuberosity, and the maxillary process of zygomatic bone in both the models. Vertically in both models, the maxillary tuberosity and all the three process of zygomatic bone showed upward movement whereas all the remaining structures showed downward displacement. Point A showed outward, backward and upward displacement in both the models (Table 2).

**Table 2:** Maxillofacial landmarks displacement (mm) after activation of quad-helix and NPE2

	Quad-helix			NPE2		
	X	Y	Z	X	Y	Z
Point A	0.003427	0.006688	−0.00881	0.003319	0.006248	−0.006096
ANS	0.004253	0.003503	0.008858	0.00391	0.003613	0.006186
PNS	0.005283	0.001837	0.012598	0.005056	0.001094	0.012617
Maxillary tuberosity	0.016233	0.00031	−0.003611	0.013944	0.000208	−0.004822
Pterygoid-hamulus	0.015453	−0.00058	0.00439	0.013264	−0.000674	0.003608
Infra-orbital rim	−0.002627	−0.003797	0.002055	−0.003208	−0.004709	0.000064
Frontal process of zygomatic bone	0.005746	−0.003224	−0.0038	0.00482	−0.004307	−0.005211
Maxillary process of zygomatic bone	0.007994	0.001986	−0.004287	0.006796	0.000902	−0.005581
Temporal process of zygomatic bone	0.001178	−0.003673	−0.006246	0.000459	−0.004526	−0.007885
Lateral nasal wall	−0.001213	−0.005725	0.004914	−0.001668	−0.006081	0.002756
Inferior nasal floor	0.001095	−0.006938	0.010252	0.000813	−0.006873	0.007878
Lateral pterygoid plate	0.016924	−0.000257	0.001381	0.0145	−0.000456	0.000907
Medial pterygoid plate	0.022838	−0.001301	0.003343	0.020164	−0.001623	0.002856

*X* transverse, *Y* sagittal, *Z* vertical, *+* indicates outward, backward and downward displacement

**Fig. 2:**
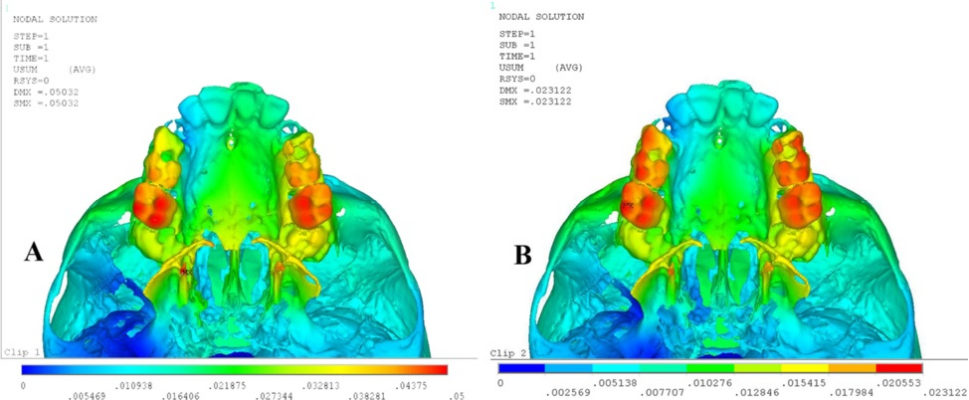
Pattern of transverse (X) displacement in the maxillary complex after the activation of **a** quad-helix and **b** NPE2

As shown in Table 3, both the groups showed opening of the mid-palatal suture in the transverse direction, and the maximum amount of dislocation was observed at the posterior region with high magnitude in quad-helix model. The anterior opening of mid-palatal suture was the same in both the groups with almost insignificant difference of displacement between both the groups. In the sagittal direction, both the models showed forward movement of all mid-palatal suture points, with a gradual decrease from the anterior to the posterior areas. Vertically, both the types had a downward movement of the mid-palatal suture points (Fig. 2).

**Table 3:** Mid-palatal suture displacement (mm) after activation of quad-helix and NPE2

	Quad-helix			NPE2		
	X	Y	Z	X	Y	Z
Point A	0.004173	−0.006665	0.008415	0.004138	−0.006013	0.005668
Point B	0.004667	−0.004224	0.012245	0.004367	−0.00415	0.010171
Point C	0.004893	−0.002638	0.012336	0.004564	−0.002295	0.011241
Point D	0.005149	−0.001793	0.012932	0.004922	−0.001159	0.012924

*Point A* point near incisive foramen, *Point D* point near palatine bone, *Points B and C* divide the A–D line into three equal parts, *X* transverse, *Y* sagittal, *Z* vertical, *+* indicates outward, backward and downward displacement

As shown in Table 4, the amounts of alveolar bone displacement in both models were greater in the posterior than in the anterior areas when viewed in transverse plane. Quad-helix showed more larger amounts of transverse expansion compared with NPE2 in the posterior region whereas the expansion in the anterior area was almost same in both the groups. Sagittally, in both the models, the alveolar bone at both the anterior and posterior area showed forward movement. Vertically, the cusp of the erupting canine showed outward, backward and extrusive movement similar to the mesiobuccal cusp of the erupting second molar which also showed outward, backward and extrusive displacement in both the appliance models.

**Table 4:** Dento-alveolar displacement (mm) after activation of quad-helix and NPE2

	Quad-helix			NPE2		
	X	Y	Z	X	Y	Z
Lingual marginal ridge of central incisor	0.005346	−0.005947	0.008094	0.005254	−0.005312	0.00549
Lingual marginal ridge of lateral incisor	0.006913	−0.004625	0.006239	0.006758	−0.004031	0.003674
Lingual marginal ridge of first permanent molar	0.017585	−0.000169	0.003302	0.015673	−0.000006	0.00233
Central incisor cusp tip	0.003598	−0.006677	0.008102	0.003688	−0.005642	0.005067
First permanent molar mesiobuccal cusp tip	0.021027	0.001396	0.005404	0.020318	0.001291	0.007759
Erupting canine cusp tip	0.00872	0.001526	0.005405	0.008523	0.002276	0.001431
Erupting second molar mesiobuccal cusp tip	0.01962	0.000524	0.002048	0.017849	0.000452	0.003237

*X* transverse, *Y* sagittal *Z* vertical, *+* indicates outward, backward and downward displacement

**Fig. 3:**
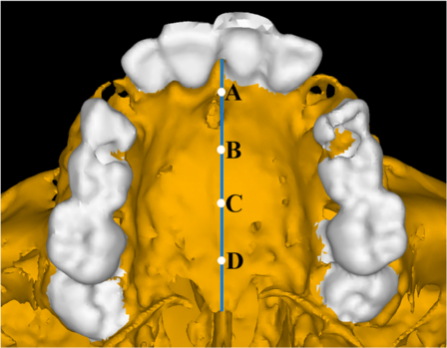
Evaluated landmarks: point *A*, point near incisive foramen; point *D*, point near palatine bone; point *B* and *C* divide the *A*–*D* line into three equal parts

**Fig. 4:**
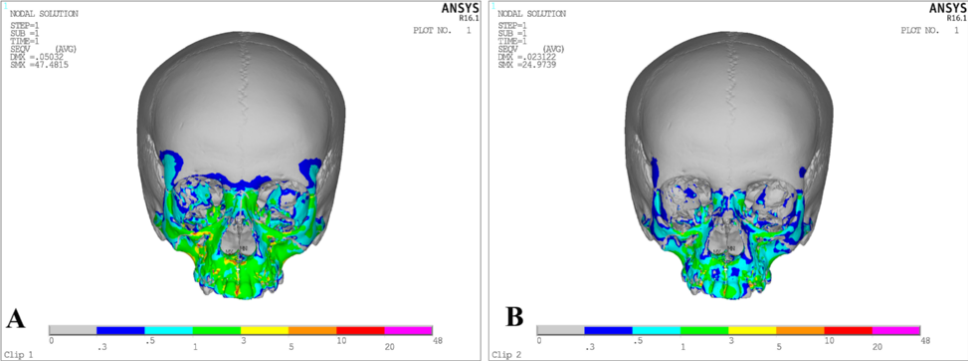
Stress distribution in the frontal view after activation of the expansion device. **a** Quad-helix. **b** NPE2

**Fig. 5:**
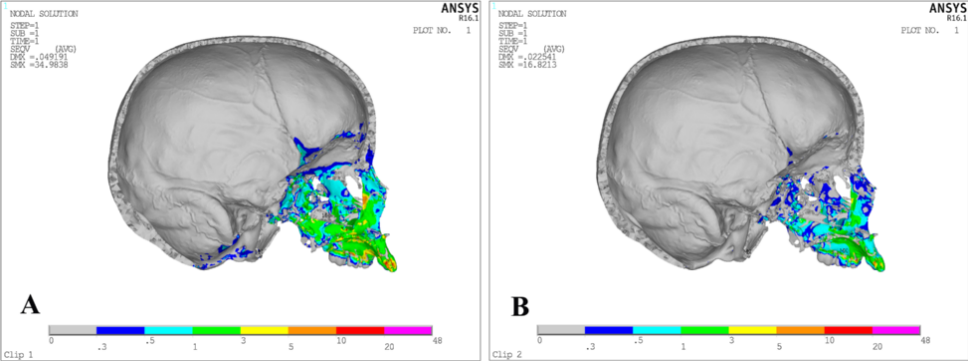
Stress distribution in the sagittal view after activation of the expansion device. **a** Quad-helix. **b**) NPE2

**Fig. 6:**
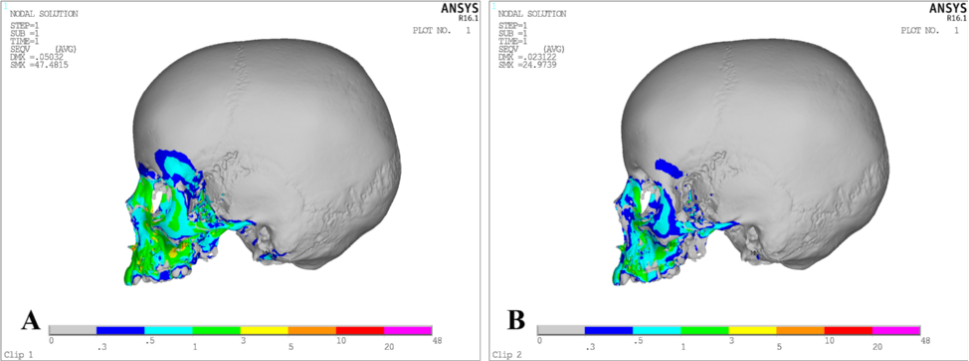
Stress distribution in the lateral view after activation of the expansion device. **a** Quad-helix. **b** NPE2

**Fig. 7:**
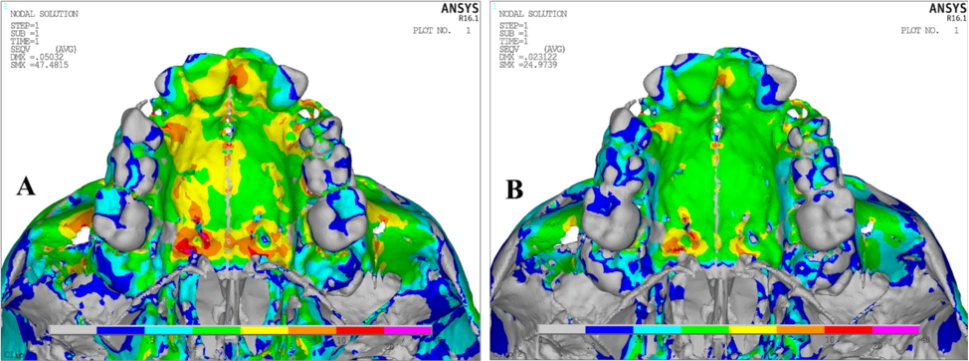
Stress distribution in the occlusal view after activation of the expansion device. **a** Quad-helix. **b** NPE2

**Fig. 8:**
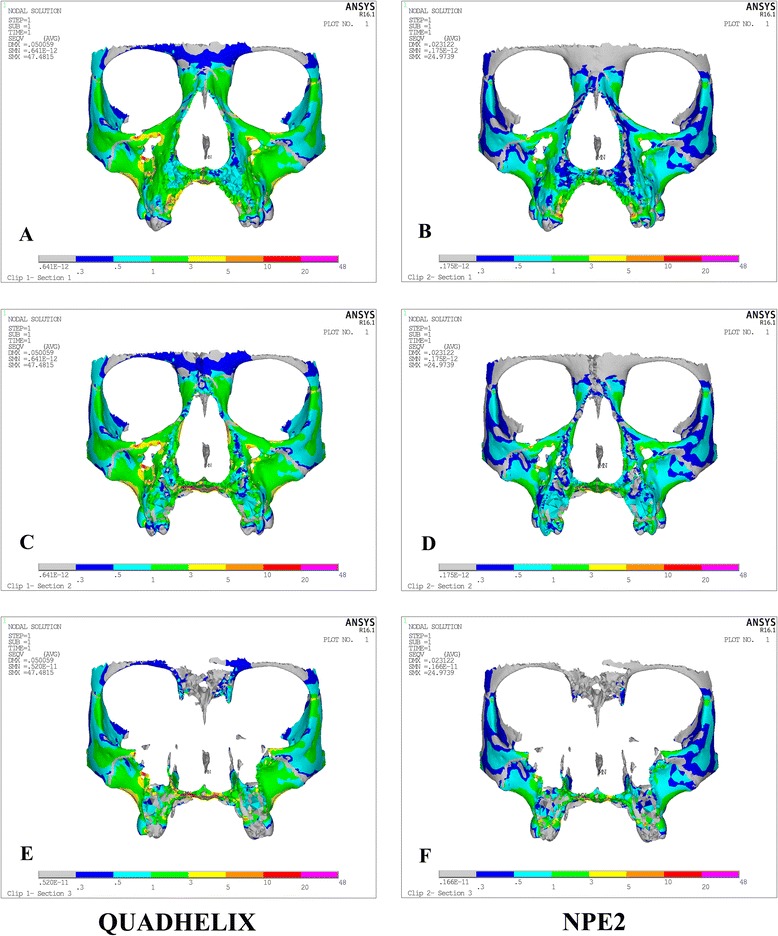
Stress distribution after activation of the quad-helix and NPE2 models in the cross-sectional view at the first deciduous molar (**a**, **b**), second deciduous molar (**c**, **d**), and first permanent molar (**e**, **f**)

Von Misses stresses as tabulated in Table 5 shows that the highest stress was recorded at the mid-palatal suture point C in both the models. Quad-helix model exhibited higher scale of stresses than did NPE2 model. The stress along the mid-palatal suture in both the models increased from anterior to posterior and then decreased rapidly near point D, i.e., near palatine bone (Fig. 3). Both the models showed similarity by representing the highest stress concentration at the fronto-zygomatic suture whereas the least stress was experienced at the fronto-maxillary suture. In exception, quad-helix model showed high stress levels around pterygo-maxillary suture whereas minimal stress around pterygo-maxillary suture was noticed in NPE2 model. The pattern of stress distribution was almost similar in both the groups, but NPE2 revealed lower magnitude stresses than quad-helix (Figs. 4, 5, 6, 7 and 8).

**Table 5:** Stress distribution (MPa) at the maxillofacial sutures and landmarks after activation of the quad-helix and NPE2 appliance

	Quad-helix	NPE2
Mid-palatal suture		
Point A	0.317823	0.317135
Point B	0.969081	0.786962
Point C	2.433233	1.896292
Point D	0.000522	0.00446
Maxillofacial landmarks		
Point A	0.434458	0.409881
ANS	0.060478	0.059042
PNS	0.022241	0.01746
Maxillary tuberosity	0.252454	0.189975
Pterygoid hamulua	0.429192	0.375754
Infra-orbital rim	1.029457	0.976392
Frontal process of zygomatic bone	0.502464	0.460146
Maxillary process of zygomatic bone	0.55113	0.509731
Temporal process of zygomatic bone	1.053598	0.9987
Lateral nasal wall	1.219208	1.165219
Inferior nasal floor	0.095343	0.100366
Lateral pterygoid plate	0.21982	0.200389
Fronto-maxillary suture	0.000932	0.000812
Zygomaticomaxillary suture	0.005582	0.005473
Fronto-zygomatic suture	0.075531	0.067953
Fronto-nasal suture	0.020907	0.017968
Zygomatico-temporal suture	0.039584	0.03841
Pterygo-maxillary suture	0.012675	0.007898
Inter-nasal suture	0.06921	0.071998
Naso-maxillary suture	0.002441	0.002493

*Point A* point near incisive foramen, *Point D* point near palatine bone, *Points B and C* divide the A–D line into three equal parts

## Discussion

In young patients, slow maxillary expansion is said to provide the maximum rate at which the mid-face sutures can adapt, with minimum tearing and haemorrhaging compared with rapid maxillary expansion [14–16]. Animal and histological studies indicate that slow maxillary expansion improves conservation of the suture and can produce a more stable result than rapid maxillary expansion [14, 15] Some clinical studies also suggest that slow maxillary expansion is more stable than rapid maxillary expansion [16]. Changes in craniofacial skeleton arising from orthodontic treatment are more complex than envisaged from two-dimensional cephalometric assessments, and so we decided to do a three-dimensional study of the craniofacial skeleton using finite element analysis.

In the present study, we witnessed skeletal changes following slow maxillary expansion, similar to those Hicks [17] had reported; according to him, substantial skeletal changes with slow maxillary expansion can be observed especially in younger children. The theory is that the main resistance to the opening of the mid-palatal suture is not the suture itself but the surrounding tissues such as the circum-maxillary structures and mid-face sutures [13]. This observation lends support to studies that noted the buttressing effect of the zygomatic processes against forces of expansion [18] supporting to evidence what we noticed in our study.

Orthopaedic force distribution after the activation of both the appliances quad-helix and NPE2 were observed to be analogous to what Chaconas and Caputo [19] stated that there was the buttressing of the maxillary tuberosity with the pterygoid plates; the sphenoid bone allowed the forces to then radiate to the base of the medial pterygoid plate from this region the forces then branched superiorly toward the malar and zygomatic bones. Specifically, the areas of the zygomatico-maxillary and zygomatico-temporal sutures were affected. The forces then radiated supero-medially toward the medial wall of the orbit and concentrated at the junction of the nasal and lachrymal bones. From our study, it is evident that the maxillary buttresses are the main areas of resistance with the forces on the maxillary molars; the stress radiates to the three main buttresses of the mid-face cranial complex: the naso-maxillary, the zygomatico-maxillary, and the pterygo-maxillary [20].

Sandikcioglu et al. [21] in his study achieved more posterior expansion of the palate; however, both the models in the present study exhibited similar results showing greater posterior dislocation of the mid-palatal suture than in the anterior region.

Our study exhibits downward and forward displacement of maxilla similar to the displacement observed by Jafari et al. [22] who studied stress distribution and displacement of various craniofacial structures following transverse orthopaedic forces. In the present study, we found backward movement of the point A which can be supported by Wertz [23] and Sandikcioglu et al. [21] who reported that the point A moved slightly backward and the ANB mostly showed high values. According to Wertz research, if it is assumed that point A does not move forward during rapid maxillary expansion, the change in the ANB angle could be a result of posterior rotation of point B [23].

During expansion, not all changes are caused by alveolar bending but are partly due to the tipping of teeth in the alveolar bone; this tipping is usually accompanied by some extrusion [24]. Similar outcomes were seen in the present study where tipping and slight extrusion of the molars were seen. Herold [25] presented greater buccal tipping in a sample treated with quad-helix. Shetty et al. [26] demonstrated tipping and extrusion of teeth following the use of NPE2.

It is witnessed in the present study that when correctly employed, the quad-helix can produce results similar to the rapid maxillary expansion and also correct all the transverse problems in the growing patients [14]. In the same way, NPE2 showed orthopaedic changes when used in mixed dentition, which is reinforced by the findings of studies that NPE2 even though being an orthodontic appliance showed orthopaedic changes [26].

The cusp of the erupting canine and the mesiobuccal cusp of the erupting second molar showed outward, backward and downward displacement indicating the speeding up for eruption which was similar to study [27] where rapid maxillary expansion was effective in treating patients in the late mixed dentition with palatally displaced canines. Baccetti et al. [28] stated that maxillary expansion is effective as an interceptive procedure to prevent final impaction of maxillary canines with palatal displacement in the early mixed dentition.

Quad-helix model showed high stress levels around pterygo-maxillary suture, whereas the stress levels were very minimal in NPE2 model which supports the findings of Donohue et al. [29] who identified that both the quad-helix and NPE2 were equally efficacious maxillary expanders; however, according to them, quad-helix appliance produced more controlled differential expansion between the first molars than NPE2 in their clinical comparison between the two and so they stated quad-helix more individually predictable in expansion.

The results of the present study using three-dimensional finite element model of a young skull provided explanation about the response of appliance activation within the bony tissues; however, finite element method has certain limitations like the results being applicable to the generated model and may not necessary apply to all individuals; moreover, clinical environment cannot be created in the model like mastication forces and patient movements; therefore, the results are for qualitative purpose for emphasizing skeletal responses of the appliances in human tissues.

## Conclusions

Quad-helix and NPE2 produced acceptable forces for orthopaedic treatment even after being an orthodontic appliance; their clinical application should be correctly planned as the effects of these appliances are largely age dependent. Both of these appliances can be used alternatively in posterior crossbite, where skeletal changes are desired at a younger age. Quad-helix showed more skeletal expansion where as NPE2 is proved to be more advantageous when dental expansion is desired. Maxillary expansion is furthermore effective in treating patients with impacted or displaced teeth because of arch length insufficiency during mixed dentition.

Although both the appliances produced almost similar amounts of maxillary expansion, from our study, we conclude that the quad-helix treatment regime is considered to be more successful if orthopaedic results are anticipated during mixed dentition period.
